# Lessons Learned from the Implementation of a Provincial Breastfeeding Policy in Nova Scotia, Canada and the Implications for Childhood Obesity Prevention

**DOI:** 10.3390/ijerph9041308

**Published:** 2012-04-16

**Authors:** Sara F. L. Kirk, Sarah Meaghan Sim, Erin Hemmens, Sheri L. Price

**Affiliations:** 1 Applied Research Collaborations for Health, School of Health and Human Performance, Dalhousie University, Halifax, NS B3H 4R2, Canada; Email: meaghan.sim@dal.ca (S.M.S.); erin.hemmens@dal.ca (E.H.); 2 IWK Health Centre, Halifax, NS B3K 6R8, Canada; Email: sheri.price@iwk.nshealth.ca

**Keywords:** breastfeeding, childhood obesity prevention, policy, supportive environments

## Abstract

Healthy public policy plays a central role in creating environments that are supportive of health. Breastfeeding, widely supported as the optimal mode for infant feeding, is a critical factor in promoting infant health. In 2005, the Canadian province of Nova Scotia introduced a provincial breastfeeding policy. This paper describes the process and outcomes of an evaluation into the implementation of the policy. This evaluation comprised focus groups held with members of provincial and district level breastfeeding committees who were tasked with promoting, protecting and supporting breastfeeding in their districts. Five key themes were identified, which were an unsupportive culture of breastfeeding; the need for strong leadership; the challenges in engaging physicians in dialogue around breastfeeding; lack of understanding around the International Code of Marketing of Breast-milk Substitutes; and breastfeeding as a way to address childhood obesity. Recommendations for other jurisdictions include the need for a policy, the value of leadership, the need to integrate policy with other initiatives across sectors and the importance of coordination and support at multiple levels. Finally, promotion of breastfeeding offers a population-based strategy for addressing the childhood obesity epidemic and should form a core component of any broader strategies or policies for childhood obesity prevention.

## 1. Introduction

The creation of supportive environments and the development of healthy public policy are two key goals of the 1986 Ottawa Charter [1]. Healthy public policy plays a central role in supporting health-enhancing behaviours, while diminishing the aspects of the environment that make it easier for individuals to engage in health-compromising behaviours [2], with the ultimate goal of shifting social norms and the physical environment so that they naturally reinforce health-enhancing behaviours [3]. Such approaches are gaining momentum as a means to address the complex factors involved in the development of chronic disease. 

Primary, upstream health begins in the gestational and early infancy periods when the foundations of health are made and life-long health behaviors are established. Breastfeeding, widely supported as the optimal mode for infant feeding, is a critical factor in promoting health. Increasing breastfeeding initiation and duration, particularly exclusive breastfeeding, is a priority for action in Canada [4]. The importance of breastfeeding is long established and offers substantial benefits for both the child and mother. Benefits for the child include protecting against illnesses and allergies, promoting mental and motor skills development, preventing childhood obesity and reducing risk factors for diabetes and heart disease in adulthood. For the mother, breastfeeding lowers the risk of breast and ovarian cancer and osteoporosis in later life, and may support post-partum weight management [4,5,6]. As research evidence reinforces the role of breastfeeding and maternal pre-pregnancy bodyweight in the development of healthy weight [7,8,9,10,11], efforts to promote breastfeeding represent an important population-based strategy for preventing childhood obesity.

In 2002, the World Health Organization set out a Global Strategy for Infant and Young Child Feeding [12]. Identifying the need for political, social and environmental commitment to realize its goals, this strategy, which was endorsed by the Fifty-fifth World Health Assembly and the UNICEF Executive Board, was designed to build on past and continuing achievements and called for an integrated comprehensive approach to its implementation. The global strategy called for exclusive breastfeeding of infants for the first six months of life, an objective that now forms the basis of all efforts to promote and monitor breastfeeding across the world. Components of the global strategy, including the International Code of Marketing of Breast-milk Substitutes [13], the Innocenti Declaration on the Protection, Promotion and Support of Breastfeeding [14] and the Baby Friendly Hospital Initiative [15], collectively fulfill an important role in the monitoring of progress towards achieving and sustaining a global culture that supports breastfeeding.

Unfortunately, increasing breastfeeding initiation and duration rates is challenging. While a significant number (90.3%) of Canadian mothers initiate breastfeeding [4], attrition rates are high and the majority will not meet the public health benchmark of exclusive breastfeeding for the first six months [16]. Although there has been an upward trend in breastfeeding initiation across Canada, rates in the Canadian province of Nova Scotia, while increasing, remain below the Canadian average [5,17,18,19]. Unfortunately, data from the Canadian Community Health Survey do not distinguish between exclusive breastfeeding and non-exclusive breastfeeding. Rates for initiation of exclusive breastfeeding in Nova Scotia, currently at around 48% of mothers with live births at the time of hospital discharge, also mask considerable variation across health districts, from 36–64% in 2009 [20]. For duration of breastfeeding, again rates in Nova Scotia lag behind the rest of Canada, but figures are disturbingly low in both cases. In 2003, only 20% of Canadian women who breastfed were exclusively breastfeeding at six months; this has risen to 26% in 2009–2010. Comparably, in Nova Scotia, just 16% of women were exclusively breastfeeding at six months in 2003, and this figure only increased to 18% in 2009/2010 [20]. 

In recognition of the importance of breastfeeding for maternal and child health and in response to the low prevalence of breastfeeding practice, public health stakeholders in Nova Scotia placed a call to action towards improving support for breastfeeding among mothers in this province through the development of a *Provincial Breastfeeding Policy* [6,21]. Released in 2005, the policy applies to the provincial government Department of Health and Wellness, District Health Authorities and all health system funded providers. The policy comprises ten directives designed to promote breastfeeding initiation and duration in Nova Scotia and to provide supportive environments for breastfeeding mothers. These directives include the need for leadership and support for breastfeeding throughout the province, along with monitoring and evaluation of the policy. 

The purpose of this paper is to describe the process undertaken to evaluate the implementation of the Nova Scotia *Provincial Breastfeeding Policy* and to highlight the key findings and lessons learned for other jurisdictions implementing similar strategies. Such an understanding can enhance the promotion of breastfeeding as an important population-based strategy for childhood obesity prevention.

## 2. Methods

This evaluation comprised focus groups held with members of provincial and district level breastfeeding committees who were tasked with promoting, protecting and supporting breastfeeding in their districts. We used a focus group guide that covered aspects of the policy directives, including leadership and communication; cross-sectoral integration; the continuum of breastfeeding support; Baby Friendly Initiative (BFI) implementation and designation; the application of the International Code of Marketing of Breast-milk Substitutes; resources and training; and monitoring and surveillance. A total of ten focus groups were held in the Spring of 2011, reflecting a total of 63 participants. The composition of each committee varied according to locally identified needs and priorities but comprised participants from a variety of backgrounds and included nursing staff, administrators, public health nutritionists, public health nurses, physicians, midwives, doulas, La Leche League leaders, business owners, government representatives and family resource centre staff. These multi-disciplinary perspectives ensured a rich discussion in all the focus groups. Additionally, we conducted telephone interviews with three senior leadership representatives to more fully capture the perceived successes and challenges of implementation, as their perspective was under-represented in the focus groups. All focus groups and interviews were digitally audio recorded, with participants providing written consent (focus groups) and oral consent (interviews) for recording. Audio-recordings were professionally transcribed by a transcriptionist working under a confidentiality agreement. Two members of the research team (EH and SFLK) independently undertook manual coding of the transcripts. Themes were then brought to the full team for review and discussion. The evaluation was reviewed by the Dalhousie University Health Sciences Research Ethics Board. 

The methodology for this evaluation followed the principles of qualitative description, in that it involved less interpretation and stayed “closer” to the data [22,23]. Focus groups are a well accepted method of data collection in qualitative descriptive research because they allow for a broad, general understanding of the topic, from multiple perspectives [22,23]. 

## 3. Results and Discussion

Five common themes were apparent across the discussion topics outlined above. These were described as an unsupportive culture of breastfeeding; the need for strong leadership; the challenges in engaging physicians in dialogue around breastfeeding; lack of understanding around the International Code of Marketing of Breast-milk Substitutes; and breastfeeding as a way to address childhood obesity. 

### 3.1. An Unsupportive Culture of Breastfeeding

The perception that there is an unsupportive culture of breastfeeding was the dominant theme across all focus groups, and arose multiple times across the discussion topics. Participants spoke of how this pervasive cultural norm impacted leadership, communication and cross-sectoral integration:

*“There’s always a big challenge in communicating with people who aren’t as involved with the [breastfeeding] work and getting that out to other people in a way that gets a hook into them and makes them pay attention”*

This unsupportive culture of breastfeeding was particularly felt to impact resource allocation. While the existence of a provincial policy had led to increased resources that were available for breastfeeding work, this was widely viewed as insufficient for the amount of work required to advance breastfeeding within the province and to challenge this unsupportive culture. Work to promote breastfeeding was often viewed as being done in addition to other roles and responsibilities, which suggests a lack of value placed on it:

*“The individuals sitting around the table are passionate about [breastfeeding] and are willing to go that extra distance and do the work even if it’s off the side of our desk or in my case as a volunteer”*

Furthermore, the resources required to ensure full implementation of the policy were also viewed as inadequate:

*“I don’t think when our system approved this policy that there was a real understanding of cost implications, the time implications and the implications in general of having that policy”*

This unsupportive culture of breastfeeding was viewed as a barrier to breastfeeding across the continuum of care, but most especially beyond six months, or when women returned to work. Specifically, the perceived lack of a supportive environment was reflected in participants’ anecdotal accounts of breastfeeding, both in the workplace and in the broader community. While participants felt that breastfeeding mothers were philosophically supported to breastfeed when they returned to work, the practical reality of needing to pump or breastfeed at work was not seen as particularly well supported:

*“I tried with my first [baby] pumping when I came back and it did not work because…when there’s work to be done, people don’t look real friendly at you if you’re running off with a pump and you’re doubling up work for somebody else while you’re gone, so if you actually get a break you could run and do it…”*

The same respondent also made the following observation, which further highlights a perceived lack of value of the breastfeeding relationship:

*“and this isn’t being judgmental either but there’s not one staff member in our facility that smokes that does not get three or four extra smoke breaks during the day and they don’t take those on their lunch breaks and their coffee breaks, they’re never questioned when they want to go out for a smoke…but granted, it doesn’t take as long to have your cigarette as it does to nurse a baby, but it’s just the whole* *thought process around it, like people don’t view that as a hindrance to your working life as much as they would to sneak off and feed baby”*

Some focus group members described their own personal experience around not feeling accepted in making the decision to breastfeed beyond six months:

*“We’ve got to change the minds, the culture has to change because even nursing at 22 months I was getting to the point, and I know I shouldn’t have been, but to the point where I felt I had to hide it. I had to hide it from my mother because she’d say ‘oh my god you’re too old to be breastfeeding this child’, or ‘she’s too old to be breastfeeding’, but you get to the point where you feel you have to hide it because it’s not normal, it’s not considered normal to others”*

For those groups that had been successful in challenging this unsupportive culture, identifying champions within other sectors was seen as crucial, as were countless hours of volunteer time contributed by community members. The need to continue to challenge the unsupportive culture for breastfeeding was seen as a main priority for action in future work around breastfeeding, with a focus on the provision of supportive environments within health and community settings.

### 3.2. The Need for Strong Leadership

Associated with the previous theme, strong leadership was felt to be a key factor in moving the breastfeeding agenda forward. In districts where senior leadership was involved in the work in some capacity, e.g., as part of a district or provincial breastfeeding committee, breastfeeding was perceived to receive greater priority. However, when this didn’t happen, there was a sense that breastfeeding was not valued. Support from senior management was often viewed as providing “lip service” and that when overall budgets were restricted breastfeeding support was reported as being one of the first areas to receive cuts:

*“[The senior leader] is very supportive but the first thing [they] said to us is ‘as long as it doesn’t cost us any money, yes, we’ll support you, just don’t ask us for a cent’”*

This was seen to undermine efforts to promote breastfeeding, and create significant challenges both to leadership and to communication:

*“…breastfeeding is the first thing to go because I don’t think they [senior leadership] really see the benefits”*

### 3.3. The Challenges in Engaging Physicians in Dialogue Around Breastfeeding

The challenges in engaging physicians in dialogue around breastfeeding was also a consistent theme across all focus groups, often coming up multiple times throughout the discussions. Key to this theme was the role of the physician as gatekeeper within the health system. If a physician is misinformed about breastfeeding, this was reported to impact the ability of a mother to initiate or sustain breastfeeding, as illustrated by the following quotes:

*“I’ve had women come and say ‘well I stopped because I was on an antibiotic and the doctor told me I couldn’t breastfeed…’”*

*“And you can’t counteract that… you can’t tell a patient your doctor is wrong, you can’t stand there and say your doctor is wrong, the patient values those physicians, those are their key person”*

The need to engage and educate physicians as well as other health professionals was raised as both a challenge and an opportunity, again touching on the previous two themes of an unsupportive culture for breastfeeding and the need for leadership:

*“The challenge is in engaging physicians, it’s engaging other departments to see they have a role to play in BFI, they don’t always see that. A mom who comes in with a five month old baby who requires surgery needs to be supported to continue to breastfeed the baby, or that perhaps knowing a mother is breastfeeding is important when mom comes into the emergency, she’s been on prescriptions that she needs to know is OK for her to take, but they need to know it too. So I think it boils down to a lack of knowledge and awareness and getting everybody on board with the same consistent and accurate messages”*

Participants referred to the success of a course on breastfeeding, called *Making a Difference* in addressing these challenges. This course is designed to address perceptions around breastfeeding and the approaches used with mothers, taking a different approach to the topic that has facilitated greater engagement and understanding of the content. However, this course, in its current format, requires a three-day time commitment, which was not seen as feasible for physicians to attend. 

### 3.4. Lack of Understanding Around the International Code of Marketing of Breast-Milk Substitutes

The aim of the International Code of Marketing of Breast-milk Substitutes is to “contribute to the provision of safe and adequate nutrition for infants, by the protection and promotion of breast-feeding, and by ensuring the proper use of breast-milk substitutes, when these are necessary, on the basis of adequate information and through appropriate marketing and distribution” [13]. The Code was the most contentious issue discussed in the focus groups and therefore represents its own theme as well as a topic of discussion. The Code was not viewed as a universally celebrated or even universally accepted document. There was a widely held belief that the Code impinges upon women’s autonomy and decision-making abilities, demonizing formula to the point of making any mother who cannot or chooses not to breastfeed feel guilty about feeding her baby formula. Worth noting is that, as a step towards BFI designation, compliance with the Code, particularly the clause which stipulates that formula must not be provided free to families, was often misconstrued by committee members to mean that any formula given to infants represented a direct violation. Several district breastfeeding committee members were able to correctly point out that the Code states in section 6.6–6.7 that providing free formula is acceptable when it is medically necessary and when the donation can be sustained for the duration of the infant’s/child’s need of it [13].

*“Mothers I don’t think understand it or even are aware that there is a Code. They think isn’t it lovely when they sign up at our local maternity stores and get samples of free formula in the mail, just in case. So their perception is O.K., this is a good thing. They don’t understand that there is a Code and what all of these implications that may mean”*

*“And I think physicians need more education and awareness of the Code. I was involved in an education session where I spoke with family practice residents and you know part of it was talking about the Code and they were very challenging about the information around the Code and you know the question is how can you make your office in the future baby-friendly and they didn’t agree that that was a necessary thing, they felt that for the most part it was a woman’s choice and they were going to support choice and, so it just spoke to me about how much more education there needed to be around that because they didn’t seem to get the bigger picture”*

Building understanding of the Code among health care providers was perceived by all district breastfeeding committees to be particularly difficult. Some committees had taken on this piece of work by hosting ‘lunch and learn’ sessions with staff; however, those committees noted that it was typically the health care providers who were already strong breastfeeding advocates who attended the sessions. Again, physicians were seen as a particularly challenging group, as many still accepted free formula and family care items, such as weigh pads and tape measures, with formula company advertisements on them. Beyond physicians, several committees, as well as the Provincial Breastfeeding Steering Committee, expressed the need for national legislation of the Code to make it a priority within the health system:

*“Canada is a signatory on this Code, but it hasn’t legislated it, so at the end of the day, that, what needs to happen is this needs to become part of federal legislation that applies across the country cause otherwise why are we a signatory on the Code?”*

Related to the Code, awareness and understanding of the process of achieving BFI designation was viewed as both a challenge and an opportunity. Several organizational challenges were mentioned, including a lack of enforcement around the steps in achieving BFI designation, along with concerns for where funding for achieving designation would come from and the often-mentioned unsupportive culture for breastfeeding. Beyond these structural challenges, there was seen to be a reluctance on the part of senior leadership to ‘buy in’ to BFI for fear of financial commitment or loss of financial support from formula producers, further highlighting the controversies associated with the Code. 

One committee member spoke of the potential for BFI designation to be part of the national accreditation process for health settings [24]:

*“I’m seeing recognition of just health promotion in general growing. But for example, we just went through accreditation but there was no real integration of BFI work into our accreditation stuff as well right, like you know it’s, we were kind of questioning so why isn’t that part of our accreditation standards?”*

Integration of BFI designation into the accreditation process would both raise awareness of the steps required for BFI designation and send a powerful message that breastfeeding was valued as a national priority.

### 3.5. Breastfeeding as a Way to Address Childhood Obesity

The final theme highlighted the importance of breastfeeding as a way to address childhood obesity. This theme was strongly expressed in two of the leadership interviews and most of the focus groups. Given the current focus on childhood obesity as a global health issue and the fact that, at the time of this evaluation, the province was in the process of developing a childhood obesity prevention strategy, there is an opportunity to increase the focus on breastfeeding as part of healthy eating in Nova Scotia. This was viewed as a way to connect breastfeeding with other provincial strategies and initiatives to address multiple agendas of relevance to population health:

*“So they talk about childhood obesity but the number one step to reducing childhood obesity is breastfeeding”...and are we really serious about that, do we really want to decrease obesity and really look at preventing diseases long-term? This is where it starts. And really support these people to continue. So how do we plan on doing that?”*

*“At least there is an interest in addressing childhood obesity and when we get the strategy to come out, I know breastfeeding is a part of it because it’s the whole healthy eating strategy that is child and youth, fruits and vegetables consumption, breastfeeding, it’s all those pieces…”*

These five themes offer important insight into the challenges and opportunities associated with the implementation of a provincial breastfeeding policy in Nova Scotia. Specifically, these themes strengthen our understanding of how the policy directives were being integrated within the broader health system and how that integration is impacting breastfeeding activities across the province, reinforcing the value of having a provincial policy. They also provide direction into the key areas that need to be addressed to create a supportive environment for breastfeeding in Nova Scotia. However, it is important to acknowledge that the themes are derived from data provided by members of provincial and district breastfeeding committees and may not be representative of health professionals who are not as involved in breastfeeding work. Despite this limitation, it is clear that a number of challenges with policy implementation remain. There was a pervasive view that senior leadership, particularly at the district level, did not fully value the importance of breastfeeding, or that the work was valued philosophically but not supported by adequate or dedicated resources for this work. This view likely reflects the societal norms that exist around breastfeeding, that still reflect an unsupportive culture. Lack of support extends beyond the health system making meaningful change extremely challenging. These norms can and should be challenged, through stronger leadership, coordination and integration of efforts to promote breastfeeding, and the provision of supportive environments for breastfeeding mothers as outlined above. This is of particular importance as the Nova Scotia government embarks on the development of a provincial childhood obesity prevention strategy [25]. Therefore, promoting the role of breastfeeding in the prevention of maternal and child obesity offers a significant opportunity to improve health across the life course.

These findings also provide a number of lessons learned for other jurisdictions working to promote breastfeeding. First, they highlight the importance of having a policy dedicated to promoting breastfeeding as a way to improve population heath. This provides accountability for actions required to implement and evaluate the policy and also highlights the importance of leadership. Strong leadership is necessary to challenge the unsupportive culture of breastfeeding and to engage health professionals, including physicians, around the importance of breastfeeding to promote health and wellbeing in mothers and their babies and provide continuity of messaging throughout the health system. Ideally, this leadership should come from multiple jurisdictions, including policy makers, health professionals and lay representatives. Second, these findings highlight the need to integrate policy with other initiatives across sectors. This means ensuring that the importance of breastfeeding is understood across multiple sectors, most particularly those that extend beyond health. While leadership is important, this needs to be accompanied by sufficient resources to challenge the unsupportive culture of breastfeeding and to embed support for breastfeeding within and beyond the health system. Third, it is important to ensure coordination and support at multiple levels. Particularly important is the need to identify ‘champions’ or ‘change agents’ within each professional group to facilitate change and move activities forward. Changing social norms is challenging and again, will require action across multiple spheres of influence, including the need for social marketing campaigns such as the one used in Nova Scotia. Finally, the childhood obesity epidemic has focused attention on ways to prevent obesity, with breastfeeding offering one potential way forward for both the mother and the child. As evidence builds for a protective effect of breastfeeding for both mother and baby, as previously outlined [4,5,6,7,8,9,10,11], promotion of breastfeeding offers a population-based strategy for addressing the childhood obesity epidemic and should form a core component of any broader strategies or policies for childhood obesity prevention. 

## 4. Conclusions

Obesity is one of the greatest challenges facing the health care system in the next century. Obesity represents an emerging public health issue globally and is the second most modifiable cause of ill health after smoking [26]. The solutions to the obesity epidemic are as complex as the problem itself and will require action across multiple levels and settings, including strong leadership, healthy public policies and greater investment in population prevention programs [27]. Promotion of breastfeeding is an important population-based strategy for addressing childhood obesity but can also enhance health throughout the lifespan. Our findings reinforce the need for greater support of breastfeeding, with the childhood obesity epidemic providing a strategic opportunity to strengthen action to promote a supportive environment for breastfeeding mothers as well as to emphasise a positive health behaviour, alongside other strategies for obesity prevention. Challenging the social norms that have created an unsupportive culture of breastfeeding, along with greater leadership from within and beyond the heath system will go a long way towards achieving what the World Health Organization, UNICEF and others set in motion over three decades ago. 

## References

[1] Ottawa Charter for Health Promotion. First International Conference on Health Promotion.

[2] 2. Lang T., Rayner G. (2007). Overcoming policy cacophony on obesity: An ecological public health framework for policymakers. Obes. Rev..

[3] Wechsler H., Devereaux R.S., Davis M., Collins J. (2000). Using the school environment to promote physical activity and healthy eating. Prev. Med..

[4] Public Health Agency of Canada (2009). What Mothers Say: The Canadian Maternal Experiences Survey.

[5] Millar W.J., Maclean H. (2005). Breastfeeding practices. Health Rep..

[6] The Healthy Eating Action Group (2005). Healthy Eating Nova Scotia Strategy.

[7] Arenz S., Rückerl R., Koletzko B., Von Kries R. (2004). Breast-feeding and childhood obesity—A systematic review. Int. J. Obes..

[8] Owen C.G., Martin R.M., Whincup P.H., Davey-Smith G., Gillman M.W., Cook D.G. (2005). The effect of breastfeeding on mean body mass index throughout life: A quantitative review of published and unpublished observational evidence. Am. J. Clin. Nutr..

[9] Harder T., Bergmann R., Kallischnigg G., Plagemann A. (2005). Duration of breastfeeding and risk of overweight: A meta-analysis. Am. J. Epidemiol..

[10] Twells L., Newhook L.A. (2010). Can exclusive breastfeeding reduce the likelihood of childhood obesity in some regions of Canada?. Can. J. Public Health.

[11] Kuhle S., Allen A.C., Veugelers P.J. (2010). Prevention potential of risk factors for childhood overweight. Can. J. Public Health.

[12] World Health Organization (WHO) (2003). Global Strategy for Infant and Young Child Feeding.

[13] World Health Organization (WHO) (1981). International Code of Marketing of Breast-Milk Substitutes.

[14] World Health Organization (WHO) (1990). The Innocenti Declaration on the Protection, Promotion and Support of Breatfeeding.

[15] World Health Organization and UNICEF (1991). The Baby-Friendly Hospital Initiative.

[16] Al-Sahab B., Lanes A., Feldman M., Tamim H. (2010). Prevalence and predictors of 6-month exclusive breastfeeding among Canadian women: A national survey. BMC Pediatr..

[17] Public Health Agency of Canada (2008). Canadian Perinatal Health Report.

[18] Chalmers B., Levitt C., Heaman M., O’Brien B., Sauve R., Kaczorowski J. (2009). Breastfeeding rates and hospital breastfeeding practices in Canada: A national survey of women. Birth.

[19] Reproductive Care Program of Nova Scotia (2000). Nova Scotia Atlee Perinatal Database, Annual Report.

[20] Statistics Canada (2011). Canadian Community Health Survey (CCHS) 1.1, 2.1, 3.1, 2007–08, 2009–10. Breastfeeding Initiation Rates.

[21] Nova Scotia Department of Health (2003). Healthy Babies, Healthy Families: Postpartum and Postnatal Guidelines.

[22] Sandelowski M. (2000). Whatever happened to qualitative description?. Res. Nurs. Health.

[23] Neergaard M.A., Olesen F., Anderson R.S., Sondergaard J. (2009). Qualitative description: The poor cousin of health research?. BMC Med. Res. Methodol..

[24] Accreditation Canada. http://www.accreditation.ca/.

[25] Province of Nova Scotia (2011). Growing Up Healthy: Developing a Childhood Obesity Prevention Strategy for Nova Scotia.

[26] World Health Organization (WHO) (2008). Obesity and Overweight.

[27] Gortmaker S.L., Swinburn B.A., Levy D., Carter R., Mabry P.L., Finegood D.T., Huang T., Marsh T., Moodie M.L. (2011). Changing the future of obesity: Science, policy, and action. Lancet.

